# A community-based treatment and rehabilitation pathway for people with mental disorders recently convicted of an offence

**DOI:** 10.1192/bjb.2024.71

**Published:** 2024-10

**Authors:** Lara Arsuffi, Kerry Ozcelik, Clare Bingham, Pamela J. Taylor, Mignon French

**Affiliations:** 1 Midland Partnership University NHS Foundation Trust, Stafford, UK; 2 Gloucestershire Health and Care NHS Foundation Trust, Gloucester, UK; 3 East London NHS Foundation Trust, London, UK; 4 Cardiff University, Cardiff, UK; 5 NHS England, London, UK

**Keywords:** Mental health treatment requirements, community sentences, mentally disordered offenders, probation, community mental health teams

## Abstract

People with mental disorders can receive treatment in the community. Some, however, fall out of services and into the criminal justice system, running the risk of imprisonment and a deteriorating mental health cycle. This editorial describes Mental Health Treatment Requirements (MHTRs), that is court-imposed sentences that enable people in the UK to access treatment in the community and divert them from short custodial sentences. MHTRs have proven successful for people with primary care mental health needs. It remains difficult to secure these sentences for people with secondary care mental health needs. Three new ‘proof of concept’ sites for secondary care MHTRs may help understand barriers and find solutions.

Mental health problems are more prevalent among those in contact with the criminal justice system than in the general population.^1,2^ Such problems include depression, anxiety, substance use and psychosis.^3^ Currently, the mental health needs of many individuals known to the criminal justice system are unmet. Untreated mental disorders can lead to risk of recidivism^4^ as well as raising risks of self-harm and suicide among prisoners.^5^ A study in Australia explored staff’s views about mental health services providing treatment to people recently convicted of an offence and living in the community. This study revealed that there are few services providing appropriate mental health treatment to this client group, due to staff’s concerns about the clients’ levels of risk, dual diagnosis/comorbidity, social needs and chaotic lives. However, as this was a small study, questions remain about the generalisability of its findings in a UK setting.^6^

Interventions designed to meet both mental health and criminal justice needs of individuals with mental disorder have been found to be associated with reductions in criminal recidivism,^7,8^ and yet there is currently a dearth of services in the community to cater for the wide-ranging needs of these people.^9^ Our focus here is on people who have at least one major mental disorder and are awaiting sentencing after conviction for a criminal offence, when their disorder is not of a nature or degree requiring in-patient treatment. A prison sentence might be an option, but the magistrate or judge is considering a community alternative, provided that mental health needs can be met within that framework.

## What are Mental Health Treatment Requirements (MHTRs) with community sentences?

The Criminal Justice Act 2003 is the legislation in England and Wales that allows for a community sentence (community order) to be tailored to meet the needs of an individual with a recent criminal conviction through specified ‘requirements’, thus facilitating future desistance from crime. This Act came into effect in 2005. Requirements may include place of residence or community service. The three community treatment requirement options are: Mental Health Treatment Requirement (MHTR), Drug Rehabilitation Requirement (DRR) and Alcohol Treatment Requirement (ATR). Before such an MHTR can be made, however, the court must have confirmation from both a probation officer and a responsible clinician (as defined under the Mental Health Act 1983) that they are willing to provide the necessary supervision and treatment respectively and the person must agree to the order. The Royal College of Psychiatrists is supportive of these principles, offering further guidance.^10^

Initially, MHTRs were not widely used by sentencers, for many reasons, including lack of awareness about them, lack of mental health screening in criminal justice settings and problems accessing suitable community mental health services.^11,12^ Other barriers included uncertainty among professionals about who should receive the order and the fact that many who would benefit from it had, alongside mental health problems, substance use problems excluding them from general adult services.^13^ Additional barriers included homelessness, as well as low clinician experience and poor service provision.^14^

Consequently, the Legal Aid, Sentencing and Punishment of Offenders Act 2012 made changes to the administration of MHTRs. These changes made it easier for individuals to be assessed for an MHTR, by removing the requirement for a section 12 registered medical practitioner and allowing qualified psychologists to complete these assessments. Subsequently, the COVID pandemic interfered with the making of these orders. Now that the pandemic restrictions have been lifted, MHTRs have become popular with the courts, and there is a national drive to use them rather than short prison sentences, as MHTRs have been found to be more effective in helping people to desist from crime.^15^

Difficulties have remained in engaging psychiatrists in this work, so NHS England, the Department of Health and Social Care, His Majesty's Prison and Probation Service and the Ministry of Justice agreed to a concept of primary care MHTRs as distinct from secondary care MHTRs. The difference lies in the nature and extent of the mental disorder(s). People eligible for a primary care MHTR will be overseen by qualified psychologists as their responsible clinician and 12 sessions of cognitive–behavioural interventions will be delivered. By contrast, secondary care MHTRs are considered for people with severe and enduring mental disorders that impair their daily functioning and these individuals would be overseen by local secondary care community adult mental health services. Generally, the team's psychiatrist would have to agree to be responsible clinician for the order to be made, but it is important to note that there is no expectation of service delivery over and above their current contractual requirements.^16^

## Are MHTRs effective as a framework for improving mental health and reducing further offending?

Research evaluating the impact of MHTRs is in its infancy, but early results are promising. Hillier & Mews,^15^ for example, explored the impact of short-term custodial sentences (<12 months) and community sentences on the recidivism rates of different groups of offenders between 2008 and 2011. They found that ‘prolific offenders’ were particularly helped, and the use of community-based sentences with an MHTR was associated with significant reductions in reoffending.

More recent analyses of outcomes after primary care MHTRs show significant improvements in mental health^17^ as well as reduction in reoffending. In other work, those under primary care MHTRs were shown to have improved quality of life.^18^ When interviewed about their experience of primary care MHTRs, offenders subject to them reported feeling listened to, accepted and more compassionate about themselves.^19^

## Where are we now with MHTRs?

Primary care MHTRs have been rolled out nationally and are currently running in many counties across England, generally delivered by stand-alone services commissioned by NHS England. By contrast, secondary care MHTRs are still underused. Many reasons are hypothesised, including (a) an increase in the number of people trying to access these services, (b) insufficient staff who can provide high-quality mental healthcare, because of high vacancies and turnover rates, and (c) the inherent challenges of partnership working, where clinicians have to work outside their ‘comfort zone’ with outside agencies,^20^ but the truth may be otherwise. Therefore, NHS England has commissioned three 24-month ‘proof of concept’ sites in England to explore the problems and find solutions. These sites will be evaluated by a team led from the University of Manchester.

The first site went live in August 2023, in Gloucestershire, staffed by a full-time qualified psychologist working across both the primary and secondary care MHTR services. The psychologist's role for the secondary care site is to act as a single point of contact when a secondary care MHTR may be appropriate, secure a responsible clinician in the local community mental health team (CMHT) or hold the order personally, also providing therapy where appropriate. To date, this service has received 19 referrals, of which 2 have been agreed by the local CMHT. The second site is London-wide, but exclusively for women. After a scoping exercise, this went live in March 2024, staffed by a 2 day per week psychologist, again acting as a single point of contact but, here, not personally offering treatment.

Preliminary reflections from these two sites have already highlighted several barriers to implementation of secondary care MHTRs, for example: (a) lack of resources in secondary care community mental health services and staff feeling overstretched; (b) rigid exclusion criteria regarding offending behaviour, risk and substance misuse, rather than thinking of risk as dynamic, contextual and manageable; (c) laborious ‘processes’ and delays in communication, which cannot match the deadlines of the court, including sentencers’ procedural timelines. Providing support and treatment for people with complex mental health needs has been linked with burnout and compassion fatigue among mental health professionals.^21^ In services that are already stretched, it is possible that such fatigue in clinicians may result in misjudgements, clinical errors and poor treatment planning,^22–24^ and it is these factors that affect the willingness of local teams to accept patients under MHTRs. Perhaps they are unaware of how working with experienced probation staff can make all the difference in enabling an offender to re-engage as a patient when health services are open to this.

The last proof of concept site to be set up is in Staffordshire. Semi-structured interviews have been conducted with 12 staff of diverse grades working for different local agencies (e.g. probation, adult social care, mental health) to establish what would help open services to secondary care MHTRs. Learning from these has led to recruitment of a full-time psychologist based in the local secondary care CMHT to deliver the tasks that have been identified as barriers to the implementation of MHTRs, including leading on assessing people who may be suitable for a secondary care MHTR, liaising with court staff and, if the order is made, steering multidisciplinary care planning and reviews, acting as the named responsible clinician on the order and liaising with probation staff. The clinical team can then focus on treatment. The University of Manchester research team evaluating the three ‘proof of concept' projects will explore all this further, as it is unlikely that teams’ strengths and weaknesses are identical across the country and, for optimal impact of any new resources, local needs must be understood. One thing that does appear clear is that, regardless of whether people eligible for MHTRs have previously been patients in the service or, more rarely, are in the catchment area but new to services, sound joint working between probation, health and the offender-patient is likely to improve the patient's motivation for recovery and desistance from offending. Being able to work on their mental health needs, rather than being excluded from community mental health services, while being under probation supervision, seems to be a sound principle of fairness and parity of esteem. Although early optimism about such integrated work^25^ has had mixed outcomes,^26^ patients have consistently expressed preference for this integrated approach to their care planning and management.^27^

No consideration of the potential value of MHTRs would be complete without considering costs in the round. These people are likely to be ‘revolving door’ patients who access accident and emergency departments and/or are sentenced to short custodial sentences that appear to achieve little more than perpetuate the offending cycle. The use of secondary care MHTRs may help break such cycles in favour of better health and social reintegration.

## Call for further discussion

It is important that people with mental disorders who offend, often at least in part because of their disorders, receive the best treatment in order to (a) reduce the number inappropriately placed in prison, (b) reduce the risk of additional morbidities and premature mortality, (c) enhance safety in our work and the wider community, and (d) alleviate their suffering and provide them with timely interventions. MHTRs are supported by the Royal College of Psychiatrists. We hope that this editorial will help engage mental health professionals from all disciplines in ongoing discussions about how to optimise implementation and delivery of MHTRs, particularly those for offenders with unequivocally specialist psychiatric needs. We are keen that these discussions take place for several reasons. First, it is possible that our struggles in getting mental health service commitment to people who need a secondary care MHTR may be reflecting the experiences of patients and their families when trying to access appropriate services before the offending. Second, we would like to hear from our psychiatrist colleagues and their community team members about how to work through the barriers to using these orders. Finally, we would like to hear from anyone who is a responsible clinician for a secondary care MHTR to learn how they negotiated the order successfully with court and probation staff, so that we can use their learning points and successes to improve the three secondary care MHTR pilots currently being trialled in England and help plan a more national solution.

## Data Availability

The preliminary reflections from the semi-structured interviews from the pilot in Staffordshire are available from the corresponding author, L.A.

## About the authors

**Lara Arsuffi** is a consultant forensic psychologist with the Midland Partnership University NHS Foundation Trust, Stafford, UK. **Kerry Ozcelik** is a principal clinical psychologist with the Gloucestershire Health and Care NHS Foundation Trust, Gloucester, UK. **Clare Bingham** is a consultant clinical psychologist with the East London NHS Foundation Trust, London, UK. **Pamela J. Taylor** is Professor of Forensic Psychiatry in the Division of Psychological Medicine and Clinical Neurosciences at Cardiff University, Cardiff, UK. **Mignon French** is Integrated Mental Health Programme Lead in the Health and Justice Central Team with NHS England, London, UK.
